# The Medium Obtained from the Culture of Hodgkin Lymphoma Cells Affects the Biophysical Characteristics of a Fibroblast Cell Model

**DOI:** 10.3390/bioengineering10020197

**Published:** 2023-02-03

**Authors:** Maura Rossi, Francesco Alviano, Barie Myrtaj, Silvia Zia, Simona Righi, Valeria Pizzuti, Francesca Paris, Barbara Roda, Andrea Zattoni, Laura Bonsi, Elena Sabattini, Claudio Agostinelli

**Affiliations:** 1 Department of Medical and Surgical Sciences, University of Bologna, 40138 Bologna, Italy; 2 Stem Sel srl, 40127 Bologna, Italy; 3 Department of Chemistry “G. Ciamician”, University of Bologna, 40126 Bologna, Italy; 4 Haematopathology Unit, IRCCS Azienda Ospedaliero-Universitaria di Bologna, 40138 Bologna, Italy

**Keywords:** lymphoma, fibroblasts, 3D model, Hodgkin lymphoma, cell culture, tumor microenvironment

## Abstract

The neoplastic Hodgkin-Reed-Sternberg (HRS) cells in Hodgkin lymphoma (HL) represent only 1–10% of cells and are surrounded by an inflammatory microenvironment. The HL cytokine network is a key point for the proliferation of HRS cells and for the maintenance of an advantageous microenvironment for HRS survival. In the tumor microenvironment (TME), the fibroblasts are involved in crosstalk with HRS cells. The aim of this work was to study the effect of lymphoma cell conditioned medium on a fibroblast cell population and evaluate modifications of cell morphology and proliferation. Hodgkin lymphoma-derived medium was used to obtain a population of “conditioned” fibroblasts (WS-1 COND). Differences in biophysical parameters were detected by the innovative device Celector^®^. Fibroblast-HL cells interactions were reproduced in 3D co-culture spheroids. WS-1 COND showed a different cellular morphology with an enlarged cytoplasm and enhanced metabolism. Area and diameter cell values obtained by Celector^®^ measurement were increased. Co-culture spheroids created with WS-1 COND showed a tighter aggregation than those with non-conditioned WS-1. The presence of soluble factors derived from HRS cells in the conditioned medium was adequate for the proliferation of fibroblasts and conditioned fibroblasts in a 3D HL model allowed to develop a representative model of the in vivo TME.

## 1. Introduction

Classical Hodgkin lymphoma (cHL) is one of the most common lymphomas. The neoplastic Hodgkin-Reed-Sternberg (HRS) cells represent only 1–10% of all cells of tumor tissue [1]; indeed, they are surrounded by an inflammatory microenvironment consisting of small CD4-positive T cells and a variable percentage of eosinophils, histiocytes/macrophages, B cells, mast cells, plasma cells, fibroblasts, mesenchymal stromal cells and endothelial cells [1,2]. A double exchange of proliferating signals between tumor cells and the microenvironment is described and this contributes to the creation of an immunotolerance state towards the tumor [3,4]. Cytokines produced by HRS cells concur to the pathogenesis of the disease both by acting as autocrine growth factors and by recruiting and sustaining the reactive infiltrate in a paracrine manner. Furthermore, cytokines produced by surrounding reactive cells contribute to the proliferation and survival of HRS cells. [5,6]. Many cytokines and chemokines, such as IL-1, IL-2, IL-3, IL-4, IL-5, IL-6, IL-7, IL-8, IL-9, IL-10, IL-12, CCL5 (RANTES), CCL17 (TARC), CCL22 and eotaxin, affect the survival of HRS cells and maintenance of its microenvironment [6,7,8,9,10,11]. Classical Hodgkin lymphoma (cHL) can be divided into four subtypes: nodular sclerosis (NSCHL), mixed cellularity (MCCHL), lymphocyte-rich (LRCHL) and lymphocyte-depleted (LDCHL). Nodular sclerosis classical Hodgkin lymphoma (NSCHL) is the most common subtype, representing 70% of cases, and is characterized by a conspicuous presence of fibroblasts and fibrosis, explained by the increased production of pro-fibrotic cytokines [12,13,14]. In the tumor microenvironment (TME) of NSCHL, the fibroblasts and the collagen-rich extracellular matrix (ECM) are involved in crosstalk with the HRS cells [5,7,8,9,14,15,16,17,18]. Hodgkin-Reed-Sternberg (HRS) cells secrete IL-7, which induces IL6 production by fibroblasts, IL-13, TGF-β and FGF that can enhance fibroblast proliferation [7,15]. They also express the IL-6 receptor and IL-7 receptor showing a significant paracrine crosstalk [14,19]; moreover, the IL-7 produced by fibroblasts can promote HRS cell growth [7,14,15,20]. In addition, HRS cells secrete IL-13 and tumor necrosis factor alpha (TNFα), which promote eotaxin and CCL5 expression in fibroblasts [8,10,14,21,22,23,24], contributing to the attraction of microenvironment cells such as eosinophils and T cells. The HL-derived cell lines have been highly useful in the in vitro preclinical studies and many cytokines have been identified in the supernatants [8,10,17,18,21,22,23,25]. In this study, we used the L-428 cell line, established from a NSCHL subtype, to exploit the rich cytokine pattern present in the medium. Indeed, the aim of this work was to study the effect of lymphoma cell conditioned medium on a fibroblast cell population and to evaluate modifications of their cell morphology and proliferation. Conditioned L-428-derived medium was used to obtain a new population of “conditioned” fibroblasts with a phenotype that could be closer to the in vivo stromal components. Differences in biophysical parameters were detected in 2D cultures by the innovative device Celector^®^, based on a label-free and field flow separation technique. Thus, fibroblast-HL cells interactions were reproduced in 3D models, creating spheroid co-cultures. We hypothesized that conditioned fibroblasts could be advantageous in developing 3D models by generating more stable and viable structures.

## 2. Materials and Methods

### 2.1. Cell Lines

In this paper, the following cell lines were used: L-428 and WS-1. The L-428 cell line was established from the pleural effusion of a 37-year-old woman with Hodgkin lymphoma (nodular sclerosis, refractory). This cell line expresses Reed-Sternberg cell markers such as CD15 and CD30. The medium used was RPMI 1640 (EuroClone, Milan, Italy) added with 1% P/S solution (streptomycin 10.000 μg/mL and penicillin 10.000 IU, Corning, Steuben County, NY, USA), glutamine (2 mM) and 10% of fetal bovine serum (FBS) (GIBCO, Life Technologies, Carlsbad, CA, USA). The WS-1 cell line is constituted by human skin fibroblasts from a 12-week healthy embryo and they grow in adherence. The medium used was FM-2 (ScienCell, Carlsbad, CA, USA) added with 1% P/S solution, (streptomycin 10.000 μg/mL and penicillin 10.000 U/mL) (ScienCell, Carlsbad, CA, USA), 1% FGS-2 (ScienCell, Carlsbad, CA, USA) and 10% FBS (ScienCell, Carlsbad, CA, USA). The L-428 cell line was bought from DSMZ-German collection of microorganisms and cell cultures GmbH; The WS-1 cell line was kindly provided by Prof. Carlo Ventura of University of Bologna. 

### 2.2. Viability Assays 

PrestoBlue cell viability assay (Invitrogen, Thermo Fisher Scientific, Waltham, MA, USA) was used to test cell viability in 2D cultures in 96-well plates. Twenty-four hours after cell seeding, the supernatants were removed and 100 μL of working solution was added for each well. After 2 h of incubation at 37 °C, the fluorescent signal was evaluated using a Perkin Elmer Wallac 1420 Victor2 Microplate Reader. A RealTime-Glo™ MT Cell Viability Assay (Promega, Madison, WI, USA) was used to test cell viability in 3D cultures 24 h after cell seeding, considering it as starting point of this assay (0 h). Luminescence was measured at different time points (0 h, 1 h, 4 h and 24 h), ending the analysis 48 h after spheroids generation, by a Perkin Elmer Wallac 1420 Victor2 Microplate Reader.

### 2.3. Conditioning of WS-1 Cell Line with Conditioned L-428-Derived Medium

The WS-1 cell line was supplemented with L-428 cell line supernatant to exploit the presence of cytokines and factors in lymphoma medium. Conditioned medium was collected after a 48 h culture of the L-428 cells. For medium harvesting, L-428 cells were seeded at a concentration of 500.000 cells/mL. The WS-1 cells were cultured in a medium containing 50% of FM-2 medium and 50% of RPMI 1640 conditioned medium for 48 h, establishing a new cell population called “conditioned” WS1 “(WS-1 COND). This cell population was compared with the non-conditioned WS-1 cell line (WS-1 CTRL), to evaluate morphological and metabolic differences. Images of WS-1 CTRL and WS-1 COND were taken with a brightfield inverted microscope Labovert FS with 4X magnification. Then, a viability assay with PrestoBlue was performed and the plate was evaluated with a Perkin Elmer Wallac 1420 Victor2 Microplate Reader.

### 2.4. Celector^®^ Characterization for WS1 CTRL and WS1 COND

Celector^®^ was used to study WS-1 fibroblasts’ physical characteristics. In particular, the analysis consisted in the comparison between “conditioned” WS-1 (WS-1 COND) and non-conditioned WS-1 (WS-1 CTRL), to investigate morphological and physical differences between the two cell populations. Celector^®^ can be defined as a cellular chromatograph patented by Stem Sel s.r.l (Bologna, Italia). The instrument analyzes and separates cells in a label-free mode based on their intrinsic physical characteristics, without additional manipulation. The instrument exploits the field-flow fractionation (FFF) principle and the patented methods Non-Equilibrium Earth Gravity-Assisted-Dynamic Fractionation (NEEGA-DF) [26,27]. The instrument consists of a fluidic system, a biocompatible capillary separation device, a micro-camera detector placed at the exit of the separation channel (MER-U3 camera, DAHENG IMAGING, Beijing, China) to monitor the elution process and a fraction collector connected to the separation device. The system generates a recorded plot of the eluted cells number (% area covered compared to the background) as a function of time (fractogram) and for frame acquisition. The software imaging data are then post-processed to obtain the geometrical features of the eluted cells as a function of time. In this work, we focused on cell diameter to obtain information on population heterogeneity during cell expansion. These geometrical features were then visualized as curves using dedicated data processing (Stem Sel Analyzer, Stem Sel s.r.l., Bologna, Italy) to obtain the average of geometrical parameter in a selected time interval (cell fraction). At first, the fluidic system is filled with sterile water, then with the coating solution (1% BSA in PBS) to cover the plastic surface of the fractionator channel and the tubes to avoid cell adhesion. Finally, there is the injection of the mobile phase (0.1% BSA in sterile PBS) to prepare the system for biological sample analysis. The flow rate is set at 1 mL/min and around 350.000 cells in 100 µL for each run were injected into the system. During the elution, cells reach a specific position across the channel thickness due to the combined action of Earth’s gravity, acting perpendicular to the flow, and opposing lift forces related to the morphological features of the sample. Due to the laminar flow of the mobile phase, cells at a specific position in the channel acquire well-defined velocities and are therefore eluted at specific times. In general, bigger and heavier cells elute before smaller and lighter cells.

### 2.5. 3D Co-Cultures of Fibroblast-Lymphoma Cells 

A forced-floating method was applied to generate 3D models. Greiner BioOne™ CellStar™ 96-Well, Cell Repellent U-Bottom Microplates were used. Conditioned WS-1 (WS-1 COND) and non-conditioned WS-1 (WS-1 CTRL) were seeded at concentration of 10.000 cells in a final volume of 100 μL of FM2 medium. L-428 and WS-1 CTRL or L428 and WS-1 COND were used to generate spheroids of co-cultures at 2:1 ratio (10.000 fibroblasts: 5.000 lymphoma cells). Cells were resuspended in a final volume of 100 μL fresh FM-2. After cell seeding, the plate was centrifuged at 50 g for 3 min to facilitate cell aggregation. Spheroids were kept in culture for 48 h and visualized with the inverted microscope Labovert FS with 4X magnification at 24 h and 48 h. To assess differences in cell viability of spheroids, RealTime-Glo™ assay was performed 24 h after cell seeding. Luminescence was measured with a Perkin Elmer Wallac 1420 Victor2 Microplate Reader at 0 h, 1 h, 4 h and 24 h.

### 2.6. Statistical Analysis 

All results were plotted using GraphPad Prism software and Microsoft Excel. Statistical analysis was performed using a two-tailed unpaired t-test, and a two-way ANOVA test was applied for Celector^®^ results. (* *p* < 0.05, ** *p* < 0.01, *** *p* < 0.001, **** *p* < 0.0001). 

## 3. Results

### 3.1. Effects of Conditioned L-428 Medium on WS-1 Cell Line Conditioning

**Figure 1:**
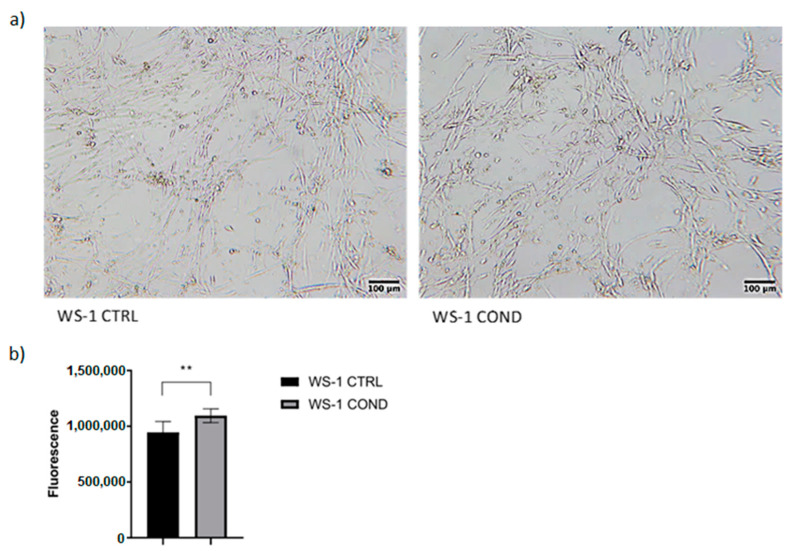
(**a**) Optical microscopy of WS-1 CTRL and WS-1 COND at 48 h after cell seeding (4× magnification). (**b**) WS-1 CTRL and WS-1 COND viability measured at 24 h with PrestoBlue assay. ** *p* < 0.01.

The culture medium of the L-428 cell line is rich in cytokines and chemokines directly involved in signaling of neoplastic microenvironment. The use of L428-conditioned medium could stimulate fibroblasts to increase proliferation, to produce extracellular matrix and to stimulate signaling towards lymphoma cells. Conditioned medium was harvested from the L-428 cell line after 48 h culture and was used in WS-1 cultures for 48 h to detect possible changes in fibroblasts proliferation and morphology, obtaining a new population of conditioned fibroblasts called WS-1 COND. WS-1 cells cultured with 100% fresh FM-2 were considered “controls” (WS-1 CTRL) and were compared to WS-1 COND. Non-conditioned WS-1 (WS-1 CTRL) showed a typical fusiform morphology. Conversely, WS-1 COND showed a different cellular morphology with an enlarged cytoplasm (Figure 1a). Furthermore, WS-1 COND showed an increased metabolism and viability compared to WS-1 CTRL by PrestoBlue assay (Figure 1b). 

### 3.2. Evaluation of WS-1 Cells Physical and Morphological Features by Celector^®^

**Figure 2:**
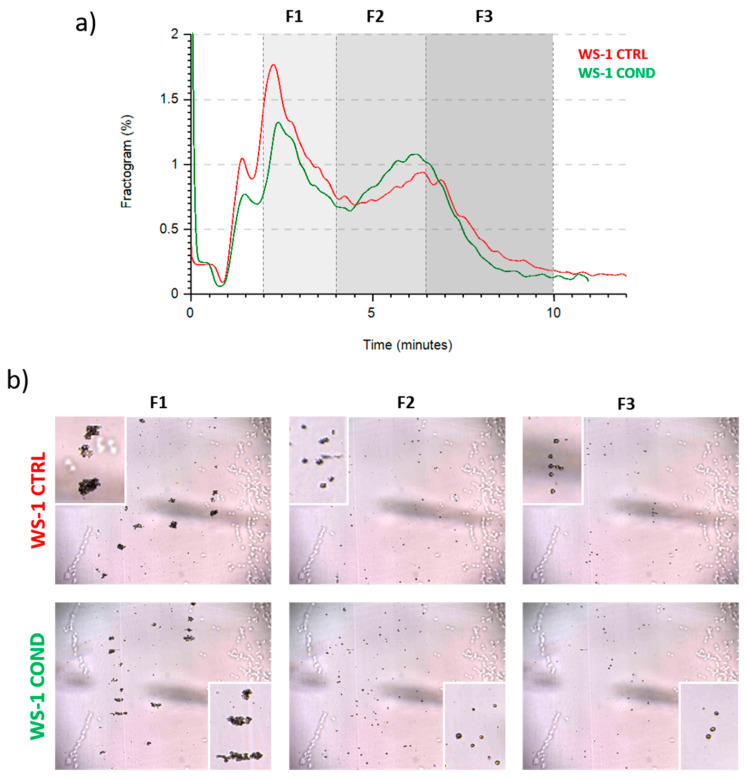
(**a**) WS-1 CTRL and WS-1 COND fractogram. (**b**) Different frames obtained from post-processing analysis of WS-1 CTRL and WS-1 COND. Cellular aggregates are particularly evident at 2 min from the beginning of measurement. In the boxes, the images show the cellular aggregates and the single cells.

**Figure 3:**
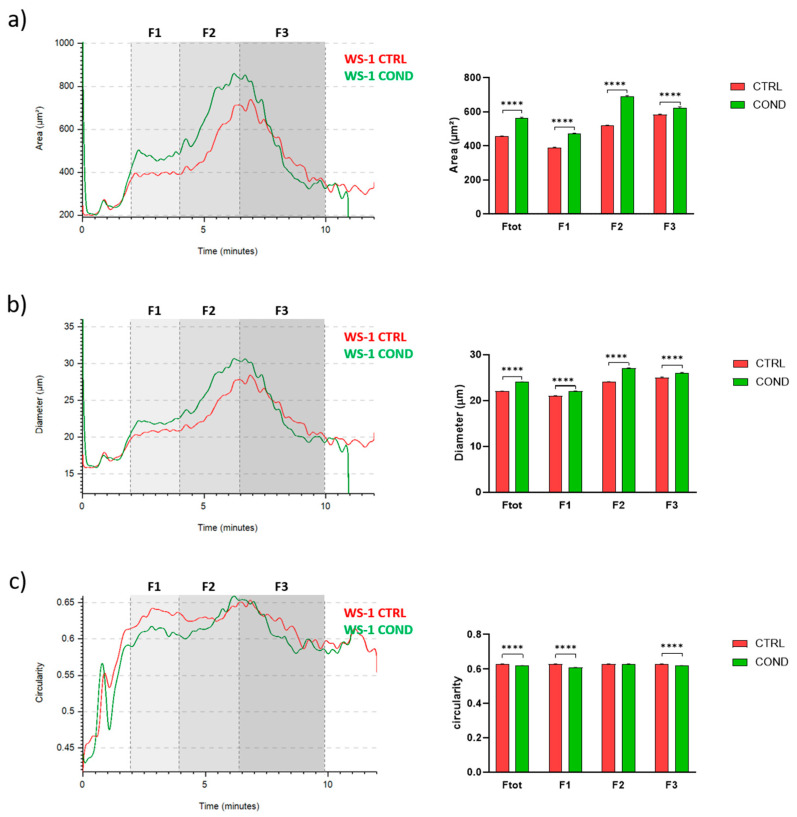
(**a**) WS-1 CTRL and WS-1 COND area profile (μm^2^) vs. time of analysis in minutes (**left panel**). Area comparison between different fractions of WS-1 CTRL and WS-1 COND (**right panel**). (**b**) WS-1 CTRL and WS-1 COND diameter profile (μm) vs. time of analysis in minutes (**left panel**). Diameter comparison between different fractions of WS-1 CTRL and WS-1 COND (**right panel**). (**c**) WS-1 CTRL and WS-1 COND circularity profile vs. time of analysis in minutes (**left panel**). Circularity comparison between different fractions of WS-1 CTRL and WS-1 COND (**right panel**). Data are presented as a mean ± standard error mean (SEM). Statistical analysis was performed using two-way ANOVA test: **** *p* < 0.0001.

To investigate the effect of conditioned medium on fibroblasts morphology, we analyzed WS-1 CTRL and WS-1 COND populations by Celector^®^, the cell chromatograph. The live fractogram, one of the outputs of the analysis, is the fingerprint of the biological sample and information on cell culture are deduced (Figure 2a). The first peak, around 1 min of analysis, named void, is characterized by cellular debris, dead cells and immediately after, from the 2nd till the 4th minute of analysis, very large cell aggregates, which do not reach the equilibrium, are soon eliminated from the channel. The main population of fibroblasts exit during the second peak, from the 4th till the 10th minute of analysis. Having a micro-camera at the outlet of the fractionator allowed the visualization of cell composition during the separation process and completed the information of the fractogram (Figure 2b). Fractogram of WS-1 CTRL and WS-1 COND did not show major differences in respect of retention time. Non-conditioned WS-1 (WS-1 CTRL) (red) presented a slightly higher intensity in the void and in the first peak, which is correlated by larger aggregates, rather than WS-1 COND. On the other hand, WS-1 COND cells are characterized by a slightly higher intensity of the second peak compared to the WS-1 CTRL. To investigate possible differences in the physical characteristics of the cells, we hypothetically divide the sample into three fractions (F1, F2, F3) to quantify cell geometrical features. The void was excluded and F1 was set from the 2nd to 4th minute, and then the main population was divided into two main fractions, the ascendent part of the curve from the 4th to the 6.5th (F2) and the descendent part from the 6.5th to 10th minute of analysis (F3). Dimensional analysis shows some differences in the cell area and diameter between the two populations of fibroblasts as seen in the overlay of the dimensional curve (Figure 3a,b, left panels). Excluding F1, which is made of cell aggerates and, therefore, single cell analysis is compromised, we focused on the main population composed of single cells, F2 and F3. Specifically, WS-1 COND cells resulted bigger in F2 compared to WS-1 CTRL, while in F3, this difference was still present around the 7th minute of analysis and then thin (Figure 3a, left panel). The same behavior was clearly seen for the parameter diameter (Figure 3b, left panel). These observations were statistically confirmed by the area and diameter values obtained for each fraction of the two cell populations (Figure 3a,b, right panels). An additional geometrical parameter measured was the cell circularity. The data presented in Figure 3c showed a slight decrease in circularity for WS-1 COND in the total population, and when F2 and F3 cells were analyzed, only F3 cells showed a difference between WS-1 CTRL and WS-1 COND cells. Non-conditioned WS-1 (WS-1 CTRL) cells were characterized by a more spherical morphology than conditioned WS-1 (WS-1 COND). These parameters confirmed the morphological differences seen in 2D cultures between the two cell populations. Indeed, the enlarged cytoplasm seen in cultures of WS-1 COND cells correlates with the increased values of area and diameter obtained by Celector^®^ measurement and its ability to discriminate and eventually sort the larger sub-population.

### 3.3. 3D Co-Cultures 

**Figure 4:**
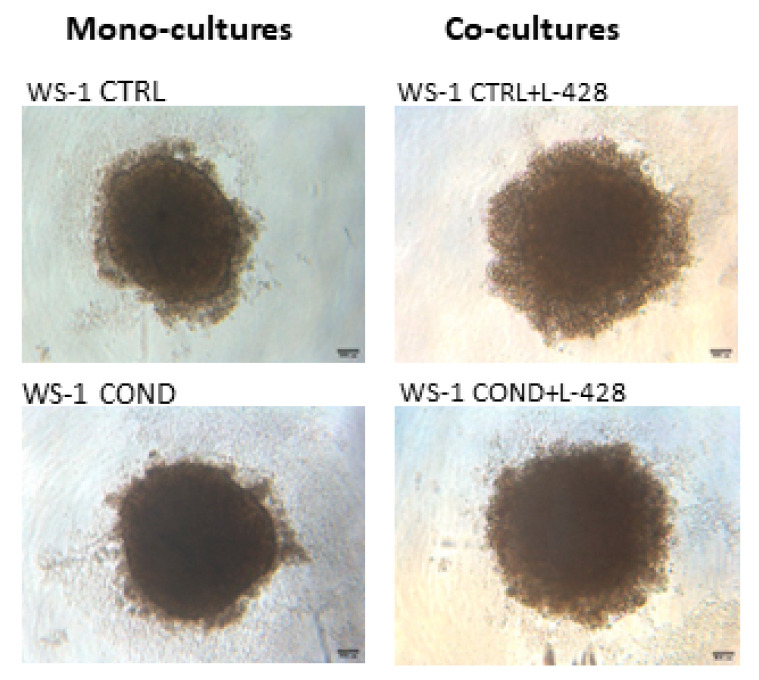
Optical microscopy of fibroblast spheroid mono-cultures of WS-1 CTRL and WS-1 COND and of fibroblast-lymphoma spheroid co-cultures of WS-1 CTRL + L428 and WS-1 COND + L428 (Ratio 2:1) at 24 h after cell seeding (4× magnification).

**Figure 5:**
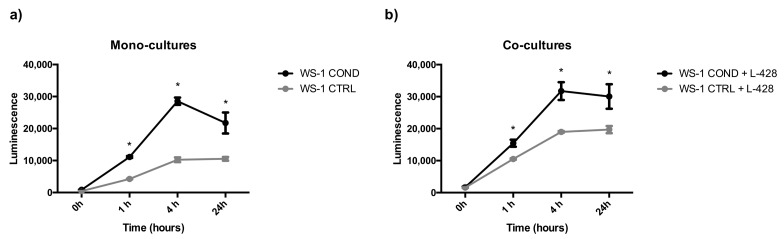
(**a**) Spheroid mono-cultures (WS-1 COND vs. WS-1 CTRL) viability assessed by RealTime-Glo assay. (**b**) Spheroids co-cultures (WS-1 COND + L-428 vs. WS-1 CTRL + L-428) viability assessed by RealTime-Glo assay. * *p* < 0.05.

Non-conditioned WS-1 (WS-1 CTRL) and conditioned WS-1 (WS-1 COND) cells were able to generate stable spheroids at 24 h after seeding in 3D plates. These structures were tightly aggregated with a dense core and well-defined borders. In the external part of the structure, there were some clusters of proliferating cells (Figure 4). To mimic the in vivo interactions and considering the differences between the two fibroblast cell populations, WS-1 CTRL and WS-1 COND cells were used for the generation of co-cultures with lymphoma cells to take advantage of the variation of WS-1 COND morphology also in 3D structures. Co-culture spheroids generated a compact structure, with a dense core and an irregular edge. Co-culture spheroids created with WS-1 COND showed a tighter aggregate than those with WS-1 CTRL and a less jagged edge (Figure 4). A RealTime-Glo viability assay was used to compare the viability of a different model of co-cultures in a 3D setting. Figure 5a,b shows the different trends for all the tested conditions at different time points. Conditioned WS-1 (WS-1 COND) showed a higher viability than non-conditioned WS-1 (WS-1 CTRL) (Figure 5a) and this behavior was also effective for their respective co-cultures (Figure 5b). Thus, the phenotype modification detected in WS-1 COND cell culture correlated with an increased metabolism and viability also in a 3D setting.

## 4. Discussion

Classical Hodgkin lymphoma (cHL) is a unique neoplasm because it is characterized by the presence of very few neoplastic cells and by a large non-neoplastic inflammatory infiltrate [1,28]. Due to the production of many cytokines, HRS cells attract and promote the expansion of different populations of immune cells, modifying their functional status to obtain survival stimuli and to switch off the antitumor immune response [29,30]. The nodular sclerosis classical Hodgkin lymphoma (NSCHL) subtype is most frequently encountered, affects adolescents, occurs frequently in mediastinal localization and is usually not Epstein–Barr virus-associated [14,28]. In this subtype, a proliferation of fibroblasts is observed, leading to sclerosing bands of birefringent collagen that confine nodular compartments [1,14]. Our understanding of the role of resting and activated fibroblasts in cancer has been greatly enhanced in recent years [31,32]. Fibroblasts associated with cancer have been termed cancer-associated fibroblasts (CAFs). In the last decade, CAFs have been considered as key components of growth, progression, invasiveness, metastasis and angiogenesis of cancer [33,34,35,36,37]. They are an integral part of fibrotic stromal support provided to cancers [34] and are a substantial source of growth factors, cytokines and exosomes [38] that can promote cancer sustenance and influence other components of TME [33]. To date, there has not been any indication that CAFs are implicated in lymphoid and hematopoietic cancers [34]. However, fibroblasts represent one of the main cellular components involved in HL, especially in the NSCHL subtype, in which IL-13R positive fibroblasts are frequently encountered around IL13 positive HRS cells, supporting the theory of a paracrine mechanism [24]. Many studies of cHL TME demonstrated that IL-13, TNF-α and TGF-β, all secreted by HRS cells, promoted fibroblast growth. These cytokines were also detected in L-428-derived medium, demonstrating the importance of HL cells-fibroblasts signaling in tumor growth [8,10,17,18,21,22,23,25]. These data led to the choice of using a conditioned medium obtained from an in vitro culture of the L-428 cell line to condition WS-1 cells for modifying cell morphology and cell proliferation, with the attempt of stimulating the ECM secretion and signaling with lymphoma cells. The L-428 cell line was chosen precisely because of its derivation from the subtype of nodular sclerosis, characterized by a large fibrotic component. The conditioned RPMI medium obtained from 48 h of L-428 cultures was tested at different concentrations in combination with FM-2 medium (data not shown). Among the tested media combinations, 50% conditioned RPMI medium was considered the optimal concentration for WS-1 cell line conditioning. It represented a source of soluble factors released from tumor cells to provide a fibroblast phenotype closer to the in vivo TME stromal component. The effect of this medium on cell morphology was evident 48 h after culturing cells in 2D plates. The conditioned WS-1 cell line (WS-1 COND) was characterized by a larger cytoplasm and a more elongated shape than the non-conditioned WS-1 (WS-1 CTRL), characterized by a typical fusiform morphology. Furthermore, WS-1 COND showed a significantly increased viability in comparison to WS-1 CTRL. These differences correlated with data obtained by an innovative field-flow fractionation device called Celector^®^. The measurement of biophysical characteristics revealed a significant increase in area and diameter of WS-1 COND compared to WS-1 CTRL, confirming the enlarged cytoplasm observed in WS-1 COND cultures by optical microscope. Moreover, the data obtained from circularity measurement could also correlate with the morphological modifications found in WS-1 COND cells. Furthermore, to increase the reliability of the in vitro model in mimicking the TME, fibroblast and HL cell interactions were reproduced in a 3D co-culture by a forced-floating method. Indeed, three-dimensional (3D) cell culture systems represent a reliable in vitro model to study tumor cell biology [39,40]. Only a few publications about 3D models in lymphomas have been published so far. Probably, the inability to produce ECM and a reduced aggregation capacity hamper the creation of a standardized and reproducible 3D model for lymphomas. Previous 3D lymphoma models created in our laboratory were characterized by weak aggregation and a loose structure that disintegrated easily during handling. Several experiments showed that lymphoma cells seeded in ultra-low attachment plates were not able to create tight spheroids, but rather a flat disk-shaped structure without compactness and poorly manageable, and this was also confirmed using different matrices (data not shown). Conversely, the generated WS-1 3D models were able to generate solid structures characterized by a dense core and delineated edges. Interestingly, WS-1 COND cells generated compact spheroids with a slightly increased size than spheroids obtained from WS-1 CTRL cells and a higher viability in 3D, with a comparable result to the 2D fibroblast cultures. Thus, fibroblasts were employed as a stromal support for the generation of WS-1 and L-428 3D co-cultures. The L-428 and WS-1 CTRL 3D co-culture exhibited a very compact structure, characterized by a dense core, suggesting that a higher number of fibroblasts than L-428 cells in the 3D co-cultures probably contributed to the generation of a more aggregate structure. The external layer showed clusters of stable cells. The use of an adapted stromal population of fibroblasts (WS-1 COND) provided co-cultures with the advantages of being more compact and with an improved vitality. Thus, the phenotype modification detected in WS-1 COND cell culture probably correlated with an increased metabolism and viability also in a 3D setting. Furthermore, fibroblasts can secrete ECM and their conditioned medium could represent a good environment rich in molecules involved in cell–cell and cell–ECM signaling. In conclusion, the presence of soluble factors derived from HRS cells in the conditioned medium was adequate for the proliferation of fibroblasts and the addition of conditioned fibroblasts in a 3D HL model allowed to develop a more representative model of the in vivo TME. 

## 5. Patents

Celector^®^ is based on a technology patented in Italy (No. IT1371772, “Method and Device to separate totipotent stem cells”), in the USA and in Canada (No. 8,263,359 US en. CA2649234, “Method and device to separate stem cells”). Stem Sel^®^ also has an Italian patent (IT1426514, “Device for the Fractionation of Objects and Fractionation Method, allowed 2016).

## Data Availability

Not applicable.
